# An exploration into “do-it-yourself” (DIY) e-liquid mixing: Users' motivations, practices and product laboratory analysis

**DOI:** 10.1016/j.abrep.2018.100151

**Published:** 2018-12-07

**Authors:** Sharon Cox, Noel J. Leigh, Taylor S. Vanderbush, Emma Choo, Maciej L. Goniewicz, Lynne Dawkins

**Affiliations:** a Centre for Addictive Behaviours Research, School of Applied Sciences, London South Bank University, 103 Borough Road, London SE1 0AA, United Kingdom of Great Britain and Northern Ireland; b Department of Health Behavior, Roswell Park Comprehensive Cancer Center, Buffalo, USA

**Keywords:** E-liquid, E-cigarettes, Vaping, Home-made liquids, DIY liquids, Harmful and potentially harmful chemicals

## Abstract

**Background:**

E-liquids are commercially available and manufactured, however some users of e-cigarettes prefer to prepare them at home (Do-it-Yourself; DIY) using individual ingredients. To date there is a paucity of research on how and why users make their own e-liquids.

**Methods:**

Forty-one European and US based exclusive users of e-cigarettes (ex-smokers) were individually interviewed. Structured interviews focused on motivations for home-mixing, practices, buying habits and broader themes around reasons for long-term vaping. We also measured nicotine and solvent concentrations and analysed 33 DIY e-liquids collected from 16 participants for nicotine, solvents, flavourings, and potentially harmful chemicals.

**Results:**

There were four main reasons for DIY: 1) economical (financial savings), 2) self-control over manufacturing process, 3) novelty, fun and 4) higher nicotine concentrations. Twelve out of 16 participants achieved nicotine concentration within 20% of their intended limit. Samples from five participants were above the EU Tobacco Products Directive's (TPD) 20 mg/ml nicotine concentration upper limit. Most samples contained more vegetable glycerine (VG) than propylene glycol (PG) and the most commonly used flavourings were dessert, *e.g.*, vanilla and caramel. Chemical analysis also revealed presence of several potentially harmful chemicals and respiratory irritants, including cinnamaldehyde, benzaldehyde, and acetoin.

**Conclusion:**

DIY may offer users of e-cigarettes a long-term affordable practical method of vaping. Recommended safety advice needs to reflect actual and fast moving user behaviour.

## Highlights

• First study investigating motivations and practices of DIY e-liquid mixing
• First known chemical analysis of e-cigarette DIY liquid
• Majority achieved nicotine concentration within 20% of the intended limit
• There is a need for research to track actual real-life user behaviour

## 1 Introduction

Electronic cigarettes (e-cigarettes) represent a ‘disruptive market’; the growth and availability of reduced risk products has changed the ways in which smokers and e-cigarette users can use nicotine. The success of e-cigarettes can in part be attributed to the related paraphernalia which makes ‘vaping’ a more pleasurable experience than either cigarette smoking or other forms of nicotine replacement therapies (Action on Smoking and Health (ASH), 2017; Notley, Ward, Dawkins, & Holland, 2018).

The rapid growth and development of the e-cigarette market means that research is not always representative of current trends. For example, newer generation devices allow a high degree of customisation by permitting changes to power (wattage) and airflow, atomiser coils can be self-made, and the availability of lower resistance atomisers coupled with high powered devices allow an increase in aerosol production (commonly referred to as sub-ohming or cloud chasing). E-liquid too can be customised by varying nicotine content, propylene glycol (PG) to vegetable glycerine (VG) ratio and proportion of flavouring, either in vape shops, or increasingly commonly, by the user him/herself (Do-it-Yourself [DIY] e-liquids).

DIY e-liquids are mixed from individual ingredients purchased from shops and/or online retailers. Kits (mixing bottles, measuring syringes) and instructions aimed at making the practice easy, and measurements precise, are widely available online and in vape shops. The extent of this practice, motivations for engagement, and the safety of the end result, however, are unknown. Users of e-cigarettes engaging in DIY e-liquids also pose a problem for researchers assessing positive (tobacco craving reduction, smoking cessation) and negative (dual use, exposure to harmful and potentially harmful chemicals) effects of e-cigarettes/e-liquid, as they introduce an additional degree of variability to an already heterogeneous category (Etter, 2014).

DIY in other consumer behaviours can be attributed to reducing cost, but more so it encompasses a way of life, being able to keep up with trends and have control over one's environment (Williams, 2004). For many this may be positively reinforcing, as hobbyist elements are a commonly cited reason for the success of vaping (Farrimond, 2017; Ward, Cox, Dawkins, et al., 2018) and DIY e-liquids could be part of this. Motivation for DIY e-liquid may be attributable to many factors, for example it can be less expensive than purchasing from a retailer. For users living in Europe, Article 20 of the European Union (EU) Tobacco Products Directive (TPD) (which came into full effect in May 2017) sets restrictions on bottle sizes (at 10 ml) and an upper nicotine concentration of 20 mg/ml may also be an added incentive.

To date, there is no empirical research on motivations for creating DIY e-liquids, whether intended nicotine concentrations reflect users' intentions, and whether such liquids contain harmful or potentially harmful constituents. A number of studies however, have analysed non-DIY commercially available e-liquids (Etter, Zäther, & Svensson, 2013; Goniewicz, Gupta, Lee, et al., 2015; Goniewicz, Hajek, & McRobbie, 2014; Goniewicz, Kuma, Gawron, et al., 2013). For example, Etter et al. (2013) found that the advertised ingredients and nicotine concentrations closely matched the product descriptions. However, they also found that impurities were above recommended safety levels although most likely below harmful levels.

The current study employed a two-step process to explore the practice of DIY e-liquids. Through structured interviews we aimed to understand i) how participants created their own e-liquids, ii) why they engage in the practice and iii) specific nicotine concentrations used. All those interviewed were asked to mail a sample of their DIY e-liquid for laboratory testing in order to explore i) intended *versus* actual nicotine and solvent concentration and ii) presence of harmful and potentially harmful constituents.

## 2 Methods

### 2.1 Participant recruitment

The study was approved by the School of Applied Sciences Ethics Committee at London South Bank University (SAS 1636) and was conducted in accordance with the ethical standards of the 1964 Declaration of Helsinki. Forty-one participants (38 males; aged 57.8 ± 4.24 (mean ± SD)) provided written informed consent (no participants were excluded). Participants were recruited from existing contacts (*i.e.,* those engaging in existing studies at LSBU) and new volunteers by promotion on social media. Inclusion criteria: any daily e-cigarette user who mixes their own liquid, aged 18 or above. Exclusion criteria: self-reported use of illicit drugs in the last 6 months including cannabis vaping.

### 2.2 Interviews with users of e-cigarettes

Individual interviews were conducted between February and March 2017 by the lead author. This timeline was after the EU (TPD) Article 20 came into force but before full-implementation. The questions were structured, based on an open-ended questionnaire with questions read aloud to participants (see Supplementary material). This style was adopted rather than a self-completed closed-question questionnaire format due to i) the dearth of research in this area, ii) to provide participants with the opportunity to give answers not anticipated by the research team and iii) to allow the researcher to request clarification and explore any unexpected answers in more depth. Interview questions were generated by the research team, in consultation with an experienced current user of an e-cigarette, based on the collective experience and knowledge of smoking, vaping and toxicology and were focused on exploring i) how people engage in the practice, ii) why they do it, and iii) the nicotine concentrations used. Specifically, 31 questions were posed relating to: Basic demographic information, current vaping status (including details on devices and e-liquids used), past smoking status, motivations for DIY e-liquid mixing (*e.g.*, why did you start making your own liquid?), purchasing (*e.g.*, where do you buy your ingredients?), mixing practices (*e.g.*, can you talk me through how you make your own liquid?) and product experiences (*e.g.*, are there any flavours that do not work for vaping?). One participant was interviewed face-to-face, three *via* telephone, and one completed the questionnaire on-line (thus questions were not read out)). The remainder (*n* = 36) were interviewed by Skype. Interviews lasted between 20 and 70 min.

### 2.3 Collection of DIY liquids for analysis

Prior to scheduled interviews, all participants were sent a stamped addressed envelope including up to four 1.5 ml Eppendorf tubes for e-liquid samples. Participants were asked to pipette a few drops of their DIY liquids into the tubes and to mail them back with a ‘recipe’ list of intended ingredients. Following interviews, all samples were sent to the Nicotine and Tobacco Product Assessment Resource (NicoTAR) at Roswell Park Comprehensive Cancer Center, in Buffalo, USA for quantitative nicotine and solvent concentration analysis as well as qualitative analysis for flavourings and potentially harmful chemicals. Upon delivery to NicoTAR laboratory, samples were stored in a dark place in controlled conditions (4 °C) prior to analyses. All samples were labelled with participant codes and laboratory technicians did not have access to information collected during interviews. In total, 33 samples were received from 16 participants (nine sent multiple samples). Twenty-six samples were received from the United Kingdom (from 13 participants), one from the Republic of Ireland (1 participant), four from Germany (1 participant) and two were received from the United States (1 participant).

### 2.4 Quantitative analysis of nicotine in DIY e-liquids

DIY liquid aliquots of 10 μl were collected directly from storage container and transferred into 1.5 ml amber glass chromatography vials with 1 ml of HPLC grade methanol (Fisher Scientific, Waltham, MA). Analyses were performed using an Agilent 7890B GC (Santa Clara, CA) as described previously^7.9^. The HP-5, 30 m × 0.320 mm × 0.25 mm (Agilent) capillary column with flow rate of helium of 7 ml/min was used. Temperature of injector was 250 °C and the detector was 250 °C, column temperature increased from 110 to 250 °C (10 °C/min) with a hold for 1 min. The injection volume was 1 μL with a split ratio of 40:1. Quinoline (100 μl of 1 mg/ml) was used as an internal standard and a calibration curve was generated in a range corresponding to nicotine concentration in e-liquids from 0 to 65 mg/ml. All samples were run in triplicate.

### 2.5 Quantitative Analysis of Solvent Volumetric Ratio (v/v) in DIY e-liquids

Fifty microliter aliquots of each e-liquid were placed in 1.5 ml chromatography vials with 1 ml of HPLC grade methanol. Analyses were performed using an Agilent 7890B gas chromatograph with a 5977A mass spectrometer. Temperature of injector, mass transfer line and ion source was 300 °C, column temperature increased from 60 to 250 °C (30 °C/min) with a hold for 10 min. The injection volume was 1 μl with a split ratio of 95:1. A SIM scan was targeted for the qualitative ions of PG (43 m/z) and VG (61 m/z). A pyridine‑*d*_5_ internal standard was used with a qualitative ions of 84 m/z. The DB-624, 30 m × 0.320 mm × 0.32 mm (Agilent) capillary column with flow rate of helium of 0.3 ml/min was used. Retention times for PG, VG and pyridine -d5 were 7.5, 10.2, and 6.1 mins, respectively. The samples were compared to a calibration curve for quantification with a v/v range from 0/100 to 100/0 PG/VG. All samples were run in triplicate.

### 2.6 Chemical component analysis of DIY e-liquids

Chromatography vials were filled with 10 μl of each sample as well as with 1 ml of HPLC grade dichloromethane (Fisher Scientific). Analyses were performed using an Agilent 7890B GC with a 5977A MS. The DB-624, 30 m × 0.320 mm × 0.32 mm capillary column with flow rate of helium of 7 ml/min was used. Temperature of injector, mass transfer line and ion source was 280 °C, column temperature increased from 110 to 250 °C (10 °C/min) with a hold for 1 min. The injection volume was 1 μl with a splitless injection. The full scan examined masses between 30 and 300 amu. Qualitative analyses of the flavored liquids were carried out using the NIST 14 MS library as well as the FFNSC 3 flavouring library. All samples were run in triplicate.

Following this analysis, each chemical was screened, by CAS number, using the Good Scents Company's chemical inventory (http://www.thegoodscentscompany.com/). This database identified if the detected compound was used as a flavouring and helped to identify hazardous chemicals (*e.g.*, harmful, irritant) from the compounds safety data sheets.

### 2.7 Statistical analysis

Interview data was categorised according to responses and presented as frequencies and means (with standard deviations). Laboratory statistical analysis was performed using Prism version 7.03 (GraphPad) or excel (Microsoft) for descriptive statics. Statistical analysis was performed on samples that were examined for nicotine and solvent concentrations. Samples were examined in one of four ways: 1. All samples were grouped together and examined using a one-way ANOVA. 2. For users who sent in two samples a Mann-Whitney non-parametric test was performed. 3. For users who sent in four samples a Kruskal-Wallis non-parametric test was performed. All experiments were performed in triplicate.

## 3 Results

### 3.1 Interview data

#### 3.1.1 Participant demographics and vaping related information

All participants were ex-smokers, and current exclusive users of e-cigarettes. The majority (28/41, 68.3%) of users reported that they prefer low nicotine concentrations (Table 1). Fourteen participants (34.2%) reported using more than one nicotine concentration in the same day, indicating an attempt to self-titrate throughout the same day, for *example*, higher nicotine concentrations in the morning and lower concentrations in the evening.

**Table 1:** Interview data results collected from 41 participants. N.B. Participants could provide more than once answer to each question.

Questions	Responses *n* = *41*
Demographic data	
Male	38 (92.7%)
Female	3 (7.3%)
Age	Mean = 57.8
	Range = 21–75 years
Years of smoking	Mean = 28.9
	Range = 7–41 years

Vaping characteristics	
Years of vaping	Mean = 3.4
	Range = 6 months–9 years
*E*-liquid used per week?	Mean = 18.2 ml
	Range = 14 ml–50 ml

Concentrations of nicotine currently used	
Low (<10 mg/ml)	26 (63.41%)
Medium (11–20 mg/ml)	10 (24.4%)
High (>21 mg/ml)	5 (12.2%)
Do you feel you need more or less nicotine than you buy in the shops?	41 (100%) no more or less

Flavours used (most popular)	
Dessert: Vanilla and Caramel	28 (68.3%)
Fruit flavours: Strawberry, Raspberry and Blackberry	24 (58.5%)

How did you find out about DIY e-liquids?	
Online forums	27 (65.9%)
Vape shops	16 (39%)
Social media (Facebook/Twitter)	16 (39%)
Friends/family	9 (22%)

Reason for starting DIY e-liquid	
Fun/novelty	41 (100%)
Higher nicotine concentration (above the available limit of 20 mg/ml or 2.0%)	25 (61%)
Financial	24 (58.5%)
Quality control	19 (46.3%)

Reasons for continuing to DIY	
Fun/novelty	38 (92.7%)
Financial	33 (80.5%)
Quality control	20 (48.8%)
Higher nicotine concentration	5 (12.2%)

Nearly all (35/41 85.4%) reported sub-ohming, though not everyone did this exclusively. Overall, nearly all participants preferred a higher VG rather than PG concentration (29/41, 70.7%). This was also evidenced in the 33 samples received with 19/33 (57.6%) e-liquids containing in excess of 60% VG; only 9 (27.3%) contained in excess of 60% PG. Of the 33 samples received, higher VG concentrations also contained lower nicotine concentrations 13/33 (39.4%).

All participants reported that dessert and/or fruit flavours worked best for vaping (flavours were more authentic and palatable over long periods of use). Interestingly, 36/41 (87.8%) used different flavours throughout the day, with some citing the use of fruit flavours (*e.g.*, strawberry, lemon) or herb/plant flavours (*e.g.*, menthol, eucalyptus) in the morning and dessert flavours (*e.g.*, vanilla, caramel) in the evening. Fifteen (36.6%) of the participants also reported using dessert flavours in the evening to replace eating sweet foods. Overall, the least popular reported flavours were tobacco, coffee and chocolate.

#### 3.1.2 DIY mixing: methods & practices

Most participants, 27/41 (65.9%), reported that they originally heard about DIY e-liquids from online forums and thirty-nine (95.1%) participants had recipes that they originally sourced from websites and use frequently; just over half (21/41, 51.2%) received recipes from vape shops. Thirty of the participants used an app (at some time) to calculate the ingredients and nicotine concentration, and five used their own personally devised spreadsheets. All but one used specially designed equipment for making their own e-liquid, *e.g.*, syringes, measuring bottles, jugs. Nearly all (38/41, 92.7%) participants stored their nicotine in a freezer (the remainder stored it in a cupboard), and most (34/41, 82.9%) stored their remaining ingredients (including flavourings) in a cupboard, often away from other food or household items.

#### 3.1.3 Motivation for home-mixing DIY e-liquids

Four common reasons for initiating DIY e-liquids were mentioned: fun/novelty, to achieve a nicotine concentration above the 20 mg/ml TPD cut-off, reduced cost and quality control (Table 1). Thirty-five participants (85.4%) expressed concern over the impending TPD changes which would affect the availability of 72 mg/ml nicotine (which is diluted and used when making DIY e-liquids). This was particularly relevant for participants from the UK and EU. Stockpiling of ingredients (82.9% said they did this), especially 72 mg/ml nicotine, was common.

### 3.2 Laboratory analysis

The following nicotine and solvent analysis is based on 33 different DIY e-liquids collected from 16 participants.

#### 3.2.1 Nicotine content

Supplementary Table 1 presents the results from laboratory testing of all samples received from participants. We examined the intended nicotine concentrations of 33 e-liquids (average 12.0 ± 8.1 mg/ml, range 3–25 mg/ml) and compared those values to the average determined nicotine concentrations (average 12.9 ± 8.1 mg/ml, range 3.1–26.8). All examined samples contained nicotine. Twelve users (75.2%) were within 20% (above/below) of their intended nicotine concentrations. Ten samples from four participants (30.3%) had higher nicotine concentrations than intended, four out of sixteen (25.0%) users produced e-liquid were >20% above/below of their intended nicotine concentration, 3/16 (18.8%) produced e-liquids that were >20% higher than intended and 1/16 (6.3%) created e-liquids that were >20% lower than intended. Of the nine DIY users that mailed multiple samples with intended different concentrations of nicotine, statistically significant differences between nicotine concentrations in samples were found for only 2 users (22.2%) (User 3 and User 4, p < 0.01). In both cases, the users had mailed four samples but only two different nicotine concentrations were found among these. Although many interviewed subjects reported that an important motivation for DIY e-liquids was to use nicotine exceeding the 20 mg/ml concentration TPD cut-off, laboratory tests revealed that only 2/15 users subject to the EU TPD (13.33%) made e-liquid that was >20% (*i.e.*, >24 mg/ml) above 20 mg/ml.

#### 3.2.2 Solvents used in DIY liquids

Laboratory testing showed that all examined samples contained both PG and VG. We examined the determined solvent volumetric ratios (average PG/VG: 43.1 ± 20.4: 57.9 ± 20.9, PG range from 10 to 80 and VG range from 20 to 90 (v/v) in 33 e-liquids sent in by the DIY users). Of the DIY users that sent in multiple samples only 2/9 (22.2%) had significantly different solvent concentrations between their own samples (User 3 and User 4, p < 0.01). In both cases each user only had two solvent concentrations among their four samples. Supplementary Table 1 presents the data for all the samples received.

#### 3.2.3 Flavourings used in DIY liquids

In the examined e-liquids, flavourings were present in all samples. The GCMS screening revealed that on average there were 9.6 ± 4.5 (Range 3–21) flavouring chemicals used in each e-liquid. Five compounds were present in all examined e-liquids: 3-hexanol, amyl alcohol, glycerol, nicotine and propylene glycol.

#### 3.2.4 Identification of potentially harmful chemicals in DIY liquids

We have identified several compounds of potential health concern (Supplementary Table 1), classified as harmful or respiratory irritants. A compound was considered harmful if it had the potential to cause death, injury or disease. Some examples of harmful chemicals include: maltol (causes damage to the liver and kidneys; Zhu, Boye, Body-Malapel, & Herkovits, 2017), benzyl alcohol (can cause bronchitis; Dart, 2004) and benzaldehyde (can cause central nervous system damage; Toxicology Data Network, 2018). A compound was considered an irritant if it causes slight inflammation or other discomfort to the body. Some examples of irritant chemicals include; 3-hexanol (known to cause dermal, ocular and respiratory irritation/damage; Hazard Identification, 2018a, Hazard Identification, 2018b, Hazard Identification, 2018c), piperonal (known to cause allergic skin reactions; Hazard Identification, 2018a, Hazard Identification, 2018b, Hazard Identification, 2018c) and acetoin (known to cause dermal and ocular irritation as well as being very flammable; Hazard Identification, 2018a, Hazard Identification, 2018b, Hazard Identification, 2018c). In addition, we identified several compounds of known health concern, acetoin (present in 8/33 of e-liquids, among 6 users) and benzaldehyde (present in 9/33 of e-liquids, among 6 users). Both compounds were present in only 2/33 samples or 1 user.

## 4 Discussion

This study investigated the practice of DIY e-liquid mixing. Hobbyist elements, such as modification of devices and tanks, is a key cited reason for vaping enjoyment (Farrimond, 2017; Notley et al., 2018; Ward et al., 2018). The interview data presented here add to this, demonstrating that creating e-liquids to personal preference, *e.g.*, taste, nicotine and PG/VG ratio, plays a role in the pleasure and hobbyist elements of e-cigarette use.

Firstly, we sought to understand how participants created their own e-liquids. Sourcing recipes online was more popular than sourcing information through vape shops. Though vape shops remain a staple for many (Brown, Beard, & West, 2018; Ward et al., 2018), our interviews highlight that for DIY purposes (pre TPD) on-line purchases were preferred, especially for participants in rural locations.

Four common reasons for initiating DIY e-liquids were reported: 1) fun/novelty, 2) higher nicotine concentration than available commercially, 3) financial, and, 4) quality control. In relation to fun/novelty, many participants explained that DIY e-liquid mixing was an extension to their other hobbies, *e.g.*, building gadgets, cooking new food recipes. Initiation of DIY e-liquids to achieve a nicotine concentration above the TPD cut-off was reported, though with the use of more powerful devices over time, there was a self-reported reduction in the nicotine concentration in their e-liquid. Financial savings were also a key reason for starting DIY e-liquid mixing. Participants suggested they could mix e-liquid at a fraction of the retail cost of commercial e-liquid. Furthermore, financial savings afforded participants the chance to experiment with a greater number of flavours. Financial motivation was intertwined with the fourth reason, perceived quality control. Those who cited this as a motivation for DIY e-liquids stated that they could make ‘high-end’ quality e-liquids for a fraction of the cost of the top brands sold in vape stores.

Furthermore, participants also felt increased satisfaction with being in control of their ingredients. This is in line with research from other consumer behaviours, showing that while financial savings are a motivating factor, this is somewhat secondary to feelings of control, pleasure and a natural extension of one's creativity and ‘life-style’ choices (Fox, 2017; Williams, 2004). Taken together, these factors appear to be positively reinforcing for the participants interviewed, providing both practical (finance) and personal (pleasure and identity) reasons to use an e-cigarette.

Reasons for continuing to make DIY e-liquids differed from the reasons given for initiating the practice. Although fun/novelty remained a key reason, reduced cost became more important, and higher nicotine concentrations than the TPD cut-off became less important. The latter may correspond to the greater frequency of use of higher powered devices during the period between initiating DIY e-liquids and being interviewed. Flavours were a key factor in all interviews; being able to produce flavours for different times of day (morning *versus* evening), occasions (palatable for all day vaping) and a number of other factors, has not been documented before. Indeed, there was indication of attempts to self-titrate using flavours. Whilst there is a strong body of evidence suggesting that nicotine self-titration can be achieved (at least partly) *via* adjusting puffing patterns in both e-cigarettes users (Dawkins et al., 2018; Dawkins, Kimber, Doig, Feyerabend, & Corcoran, 2016) and smokers (Ashton, Stepney, & Thompson, 1979; Russell, Jarvis, Iyer, & Feyerabend, 1980), how flavours may impact puffing patterns, usage styles and nicotine delivery has only recently been explored (St Helen, Shahid, Chu, & Benowitz, 2018). Furthermore, there was variation in PG/VG ratio both between and within participants. How these variations might influence puffing patterns and usage styles with any implications for exposure to potentially harmful chemicals or maintaining abstinence from smoking, requires further exploration.

In relation to nicotine concentrations, along with a range of flavours, some participants varied their nicotine concentrations depending on time of day (morning for stronger concentrations, night time for lower concentrations) and other occasions. For example, higher nicotine concentrations were associated with evenings out or before periods of abstinence (*e.g.*, flights, work environments). Although there is evidence that users of e-cigarettes switch to lower nicotine concentration over time (Etter, 2016), how and why users vary nicotine liquid concentrations within a day deserves greater attention, especially in relation to developing tailored health messages which can enable people to remain abstinent from cigarette smoking. Despite the fact all of our participants were exclusive users of an e-cigarette, and this was a small sample, such levels of variation in user behaviour by flavour and nicotine concentration makes standardising ‘typical’ usage patterns in which to inform laboratory based studies a difficult task.

The majority of participants reported using low nicotine concentrations (categorised as below 10 mg/ml) and many also sub-ohmed. Only one participant used a high concentration of nicotine and also sub-ohmed. To date, there is paucity of research on how sub-ohming affects users' e-liquid preferences, behaviour, and subsequently, the production of toxins in the vapour. Although sub-ohming is commonly related to the young adults' practice of ‘cloud-chasing’, there was no mention of cloud chasing or vape tricks (though not explicitly asked) in this study and the average participant age was 57 years. This suggests that sub-ohming, or any other hobbyist elements, are not explicitly related to a specific sub-group of younger users of e-cigarettes (similar findings have been observed by Ward et al., 2018).

This study was conducted at a time when participants could purchase 72 mg/ml nicotine (which is diluted and used to make DIY liquids). Many participants expressed concern that vaping would become more expensive and less pleasurable without the range of products previously available, although nobody stated that they would resume smoking. Participants' expressed concern that black market websites selling high strength nicotine would stand to profit from the TPD upper nicotine concentration legislation. Participants reported receiving regular targeted emails and social media messages from black market vendors who were actively capitalising on the impeding market changes. Of note, all participants were long-term users of e-cigarettes, having given up smoking, and had no intention of quitting vaping; many noted that they did not view e-cigarettes as a quitting aid but instead a reduced risk alternative to smoking. DIY e-liquid mixing appeared to play a significant role in the longer-term enjoyment for those interviewed who reported no desire to quit vaping; how the restriction on sales of 72 mg/ml nicotine will impact this enjoyment remains to be determined.

The majority of DIY users were able to achieve their intended nicotine concentrations ±20% demonstrating that DIY users are generally capable of creating nicotine e-liquids to a similar level of accuracy as commercial products. Additionally, the users that had inaccuracies in their nicotine dilutions were those who sent multiple samples. We found that these DIY users were consistently incorrect. We suspect these discrepancies to be a result of one of the following reasons; 1) lack of accuracy or knowledge surrounding their measurement devices (*e.g.*, syringes, beakers); 2) incorrect calculations from the app/spreadsheet being used; 3) lack of mixing of stock e-liquid before shipment for laboratory analysis. Nevertheless, even in these few participants, although the intended *versus* actual nicotine concentration discrepancy was large in percentage terms, in mg/ml, the difference was small (up to ±3 mg/ml difference).

Participants who sent in e-liquids used an average determined nicotine concentration of 12.9 mg/ml with an average intended concentration of 12 mg/ml. These results are consistent with the interview data showing that DIY users are involved in the process for fun/novelty rather than for trying to create e-liquids with extremely high nicotine concentrations. The availability of newer, higher powered devices with sub-ohm coils (commonly used in our sample) allows more efficient vaporisation of e-liquid and in turn, necessitates lower nicotine concentration e-liquids. Thus product evolution is likely a key driver of the reduced desire to produce high nicotine concentration e-liquids.

Lastly, these findings are in line with previous studies on commercially manufactured e-liquids, which have also consisted of a wide variety of flavouring chemicals (Hutzler et al., 2014; Kavvalakis, Stivaktakis, Tzatzarakis, et al., 2015; Lisko, Tran, Stanfill, Blount, & Watson, 2015; Tierney, Karpinski, Brown, Luo, & Pankow, 2016). In the current study, on average we detected 9.5 flavouring chemicals in DIY e-liquids. Many of the flavouring chemicals detected were previously reported in commercial products, including ethyl maltol, ethyl vanillin, acetoin, menthol vanillin, benzaldehyde, anisaldehyde, and benzyl alcohol. Therefore, our sample of DIY e-liquids did not appear to introduce any greater variance in chemicals than commercially available e-liquids.

There are some limitations to this study, our data is cross-sectional and the sample size is small, we received only 33 e-liquid samples from 16 participants. We recruited *via* social media, meaning that we could have elicited responses from those who are most keen to be involved in research and also those heavily invested in vaping.

To conclude, this is the first study to examine DIY uses, behaviour and e-liquid constituent preferences. Although DIY users have the potential to bypass government regulations, (*e.g.,* TPD), the majority of our sample were not producing e-liquids that differ significantly from commercial manufactured products and the most common reason for continuing the practice was the fun/novelty value. This is a relatively new practice/e-cigarette trend, and along with sub-ohming, more research is required to document the prevalence, practice and effects of such trends and the transiency of their nature.

The following are the supplementary data related to this article.

Appendix A (For interviewer to ask participants) Appendix A

Supplementary Table 1 Nicotine and solvent concentrations results collected from 16/41 participants in 33 samples. Supplementary Table 1

## Funding

No funding was received.

## Declaration of interest

SC has provided expert consultancy services to UK life insurers on smoking cessation and reduce risk products prevalence rates.

NL, EC, TV have no conflicts of interest to declare.

MLG has received a research grant from Pfizer and serves on an advisory board to Johnson & Johnson, manufacturers of smoking cessation medications.

LD has conducted research for independent electronic cigarette companies. These companies had no input into the design, conduct or write up of the projects. She has also acted as a consultant for the pharmaceutical industry and as an expert witness in a patent infringement case (2015).
