# Supplementary material for: An exploration into “do-it-yourself” (DIY) e-liquid mixing: Users' motivations, practices and product laboratory analysis
Source: Addict Behav Rep. 2018 Dec 7;9:100151. doi: 10.1016/j.abrep.2018.100151 (PMC6542371; doi:10.1016/j.abrep.2018.100151)
Supplement: Appendix A — (For interviewer to ask participants) [file mmc1.docx]

**Appendix A (For interviewer to ask participants)**

1. How long have you been vaping?
2. What type of device do you use? Please name the brand, model, tank etc.
3. Do you sub-ohm?
4. How long have you been making your own e-liquid?
5. Why did you start making your own e-liquid?
6. Have these reasons changed? Are there any other reasons why you make your own liquid?

e.g. for personal use? For other people?

1. How did you find out about making your own e-liquid?
2. How often do you make your own e-liquid?
3. How much do you use in one week?
4. What level of nicotine do you use?
5. Do you feel you need more or less nicotine than the products you buy in the shops?
6. Do you ever (still) buy ready-made liquid?
7. What flavours do you use (list them here):
8. Do you have a favourite flavour?
9. What are the ingredients?
10. Which flavours work best?
11. Have you tried any that were not so good? Why were they bad?
12. How much do you make in one batch?
13. Where do you store the ingredients and the end results?
14. How long do you store it for?
15. Where do you get the nicotine from? Is it concentrated or pure?

How do you calculate and adjust nicotine concentration in your liquid?

1. Where do you get the PG/VG from?
2. Where do you get the flavourings from?
3. Do you have a favourite supplier? Why are they a good supplier?
4. Have you ever bought any products that did not seem right to you? Or poor quality? What was wrong with these products?
5. How do you get your recipes?
6. Please describe in your own words how you prepare your liquid
7. Have you ever experienced any side effects or unpleasant taste from the products you have made?
8. Have you ever felt that you made your liquid too strong?
9. Is there anything else you would like to tell me about?
