# Supplementary material for: An exploration into “do-it-yourself” (DIY) e-liquid mixing: Users' motivations, practices and product laboratory analysis
Source: Addict Behav Rep. 2018 Dec 7;9:100151. doi: 10.1016/j.abrep.2018.100151 (PMC6542371; doi:10.1016/j.abrep.2018.100151)
Supplement: Supplementary Table 1 — Nicotine and solvent concentrations results collected from 16/41 participants in 33 samples. [file mmc2.docx]

| **Participant** | **Sample Code** | **Total Compounds Detected** | **Intended Nicotine Concentration (mg/ml)** | **Average Determined Nicotine Concentration (mg/ml) / % Different from Intended** | **PG/VG Ratio (v/v)** | **Total Number of Flavouring Chemicals Detected** | **Flavouring Chemicals Classified as Respiratory Irritants^#^** | **Flavourings Chemicals Classified as Potentially Harmful Chemicals^#^** | **Other Potentially Harmful Chemicals** |
| --- | --- | --- | --- | --- | --- | --- | --- | --- | --- |
| **United Kingdom** | | | | | | | | | |
| 1 | 1A | 14 | 18 | 21.0 (+14%) | 80/20 | 6 | 3-Hexen-1-ol, Acetoin,  Diacetone alcohol (DAA), γ‑Decalactone | Methyl propionate | Benzenemethanol, α.,4-Dimethyl-butyric acid,  Nicotyrine,  Valeric acid |
| 3 | 3A | 12 | 18 | 20.4 (+12%) | 65/35 | 7 | Terpineol | Benzyl alcohol, Cinnamaldehyde, Ethyl maltol, Eugenol,  Maltol | Nicotyrine |
|  | 3B | 20 | 16 | 17.5 (+9%) | 70/30 | 11 | 3-Hexen-1-ol, γ‑Decalactone, Methyl cinnamate | Ethyl maltol,  Ethyl menthane carboxamide, Maltol,  Methyl propionate, Pineapple ketone, Vanillin | 2-Methyl butyric acid,  Butyric acid, Nicotyrine, Triacetin,  Triethyl citrate,  Valeric acid |
|  | 3C | 19 | 6 | 7.2 (+17%) | 30/70 | 13 | Acetoin, Anisaldehyde, γ‑Octalactone, Piperonal,  Sulfurol | Anisyl alcohol, Benzyl alcohol, Ethyl vanillin, Guaiacol, Maltol, Vanillin | Butyric acid, Nicotyrine |
|  | 3D | 12 | 6 | 6.2 (+3%) | 35/65 | 7 | Sulfurol | Benzyl alcohol,  Ethyl maltol,  Ethyl vanillin,  Maltol,  Vanillin | Valeric acid |
| 4 | 4A | 19 | 6 | 6.1 (+2%) | 25/75 | 11 | Anethole, Anisaldehyde,  Eucalyptol, Linalool,  Menthol,  Terpineol | Benzaldehyde (and its derivatives),  Estragole,  Ethyl maltol | Anethol,  Carvone,  Foeniculin,  Nicotyrine, 2,6‑Diethylpyrazine |
|  | 4B | 21 | 10 | 10.1 (+1%) | 20/80 | 12 | α-Ionone,  Anethole, β‑Damascone, Ethyl butyrate,  Eucalyptol, Linalol,  Menthol,  Piperonal | Estragole,  Maltol,  Raspberry ketone,  Vanillin | Anethol, Benzenemethanol, α4-SDimethylbutyric acid,  Nicotyrine |
|  | 4C | 16 | 18 | 19.7 (+9%) | 55/45 | 10 | Anisaldehyde, Piperonal | Anisyl alcohol, Benzaldehyde (and its derivatives),  Ethyl maltol,  Ethyl vanillin, Hydroxycoumarin,  Maltol,  Vanillin | Nicotyrine |
|  | 4D | 11 | 7 | 7.3 (+4%) | 25/75 | 5 | Anisaldehyde, Piperonal | Benzaldehyde (and its derivatives),  Ethyl menthane carboxamide,  Vanillin | Nicotyrine |
| 5 | 5A | 21 | 6 | 5.7 (-5%) | 30/70 | 15 | Anisaldehyde, Ethyl butyrate, γ‑Nonalactone, γ‑Octalactone, Piperonal | Anisyl alcohol,  Benzaldehyde (and its derivatives),  Benzyl alcohol,  Ethyl maltol,  Ethyl vanillin,  Maltol,  Vanillin | Butyric acid,  Cyclotene,  Valeric acid |
| 7 | 7A | 32 | 6 | 3.2 (-88%) | 25/75 | 21 | 3-Hexen-1-ol,  Acetoin,  Acetylpropionyl,  Anisaldehyde,  γ‑Decalactone,  γ‑Octalactone,  Methyl cinnamate,  Piperonal,  Sulfurol | Anisyl alcohol,  Benzaldehyde (and other derivatives),  Ethyl maltol,  Ethyl vanillin,  Guaiacol, Hydroxycoumarin,  Maltol,  Methyl propionate,  Pineapple ketone,  Vanillin | 2-Methyl butyric acid,  Butyric acid,  Cyclotene,  Nicotyrine,  Pentanoic acid,  5-Hydroxy-2,4-di-t-butylphenyl ester,  Triethyl citrate |
|  | 7B | 23 | 6 | 3.1 (-94%) | 20/80 | 16 | Acetoin, γ‑Decalactone | Benzaldehyde (and other derivatives),  Benzyl alcohol,  Ethyl maltol,  Ethyl vanillin,  Hydroxycoumarin,  Maltol,  Pineapple ketone,  Raspberry ketone,  Vanillin | 2-Methyl butyric acid,  Butyric acid,  Cyclotene,  Pentanoic acid,  5-Hydroxy-2,4-di-t-butylphenyl ester,  Valeric acid |
| 8 | 8A | 15 | 3 | 3.9 (+23%) | 15/85 | 8 | Methyl anthranilate | Benzaldehyde (and other derivatives),  Benzyl alcohol,  Ethyl maltol,  Maltol,  Pineapple ketone,  Raspberry ketone,  Vanillin | Pentanoic acid,  5-Hydroxy-2,4-di-t-butylphenyl ester,  Triethyl citrate |
|  | 8B | 10 | 3 | 3.9 (+23%) | Oct-90 | 5 |  | Benzyl alcohol,  Ethyl maltol,  Ethyl vanillin, Hydroxycoumarin,  Vanillin |  |
| 9 | 9A | 9 | 5 | 5.8 (+14%) | 20/80 | 3 |  | Benzyl alcohol,  Ethyl vanillin,  Vanillin | Nicotyrine |
| 10 | 10A | 11 | 7 | 7.7 (+9%) | 20/80 | 5 | Acetoin,  γ‑Nonalactone | Benzyl alcohol,  Toncarine,  Vanillin | Nicotyrine |
| 11 | 11A | 11 | 24 | 26.8* (+10%) | 35/65 | 5 | Piperonal | Benzaldehyde (and its derivatives),  Benzyl alcohol,  Ethyl vanillin,  Vanillin | Nicotyrine |
|  | 11B | 19 | 24 | 25* (+4%) | 45/55 | 11 | Anethole,  Anisaldehyde,  Eucalyptol,  Linalool,  Menthol,  Terpineol, | Estragole,  Eugenol,  Pulegone | Anethol,  Foeniculin, Nicotyrine,  2,6‑Diethylpyrazine,  Zingerone |
| 12 | 12A | 12 | 6 | 6.8 (+12%) | 20/80 | 6 |  | Cinnamaldehyde,  Ethyl vanillin, Eugenol,  Vanillin | Nicotyrine,  Zingerone |
| 13 | 13A | 17 | 5 | 8.4 (+40%) | 65/35 | 10 | Anethole,  Anisaldehyde,  Eucalyptol,  Linalool,  Menthol,  Piperonal | Maltol,  Raspberry ketone,  Vanillin | Anethol,  Butyric acid,  Nicotyrine |
|  | 13B | 13 | 5 | 7.8 (+36%) | 70/30 | 8 | γ-Decalactone,  Methyl isobutyrate,  Terpineol | Benzyl benzoate,  Maltol | Butyric acid,  Nicotyrine,  Valeric acid |
|  | 13C | 24 | 5 | 8.2 (+39%) | 65/35 | 16 | 3-Hexen-1-ol, Anethole, γ‑Damascone,  Eucalyptol,  γ‑Decalactone,  Linalool,  Menthol,  Methyl anthranilate,  Methyl cinnamate | Estragole,  Ethyl menthane carboxamide,  Maltol,  Phenylethyl alcohol,  Vanillin | 2-Methyl butyric acid,  Anethol,  Butyric acid,  Nicotyrine,  Triacetin |
|  | 13D | 13 | 5 | 9.3 (+46%) | 65/35 | 7 | Γ‑Decalactone, Linalool | Benzyl alcohol,  Eugenol,  Raspberry ketone,  Vanillin | Butyric acid,  Nicotyrine |
| 14 | 14A | 22 | 7 | 7.3 (+4%) | 55/45 | 14 | 3-Hexen-1-ol,  Anethole,  Ethyl butyrate,  Eucalyptol,  γ‑Decalactone,  Linalool,  Menthol,  Methyl anthranilate, Methyl cinnamate | Ethyl menthane carboxamide, Maltol,  Phenylethyl alcohol | 2-Methyl butyric acid,  Anethol,  Nicotyrine, Triacetin,  Valeric acid |
| 15 | 15A | 20 | 9 | 13.5 (+33%) | 75/25 | 13 | α-Ionone,  Anethole,  β‑Damascone, Eucalyptol,  γ‑Decalactone, Menthol,  Methyl anthranilate,  Piperonal | Ethyl menthane carboxamide,  Maltol,  Raspberry ketone,  Vanillin | Anethol,  Butyric acid,  Nicotyrine |
|  | 15B | 26 | 12 | 16.6 (+28%) | 75/25 | 17 | 3-Hexen-1-ol,  α‑Ionone,  Anethole,  β‑Damascone, Ethyl butyrate,  Eucalyptol, γ‑Decalactone,  Linalool,  Menthol,  Methyl anthranilate,  Methyl cinnamate | Ethyl menthane carboxamide, Maltol,  Phenylethyl alcohol | 2-Methyl butyric acid,  Anethol, Benzenemethanol, α4-Dimethyl-butyric acid,  Nicotyrine,  Triacetin,  Valeric acid |
| **Republic of Ireland** | | | | | | | | | |
| 2 | 2A | 23 | 8 | 6.8 (-18%) | 15/85 | 16 | Acetoin,  Acetylpropionyl,  Anisaldehyde,  Ethyl butyrate,  γ-Nonalactone,  γ-Octalactone,  Piperonal,  Sulfurol | Anisyl alcohol,  Ethyl maltol,  Ethyl vanillin, Guaiacol,  Maltol,  Vanillin | Butyric acid,  Nicotyrine |
| **Germany** | | | | | | | | | |
|  | 6A | 12 | 24 | 22.9* (-5%) | 40/60 | 6 | Piperonal | Ethyl maltol,  Ethyl vanillin,  Maltol,  Vanillin | Nicotyrine |
| 6 | 6B | 12 | 24 | 22.5* (-7%) | 40/60 | 5 | Acetoin,  α-Ionone,  β‑Damascone,  Ethyl butyrate | Pulegone, Raspberry ketone | Nicotyrine |
|  | 6C | 11 | 24 | 23.7* (-1%) | 40/60 | 4 | Diacetone alcohol,  γ-Decalactone,  Methyl isobutyrate | Vanillin | Butyric acid,  Nicotyrine |
|  | 6D | 15 | 24 | 21.6 (-11%) | 40/60 | 8 | 3-Hexen-1-ol,  Menthol,  Methyl anthranilate | Benzyl alcohol,  Ethyl vanillin,  Maltol,  Vanillin | 2-Methyl butyric acid,  Carvone,  Nicotyrine |
| **United States** | | | | | | | | | |
| 16 | 16A | 10 | 25 | 25.4* (+2%) | 50/50 | 4 | γ‑Nonalactone | Benzyl alcohol,  Toncarine,  Vanillin | Nicotyrine |
|  | 16B | 18 | 25 | 25.7* (+3%) | 50/50 | 11 | Ethyl butyrate,  Piperonal,  Sulfurol, | Benzaldehyde (and its derivatives),  Benzyl alcohol,  Benzyl benzoate,  Ethyl maltol,  Ethyl vanillin,  Maltol,  Vanillin | Nicotyrine,  Triacetin |
| **Average±SD** | | 16.5±5.6 | 12.0±8.1 | 12.9±8.1 | PG: 43±20 VG: 58±21 | 9.6±4.5 | Average:  4 chemicals | Average:  4 chemicals | Average:  2 chemicals |
